# DNA Methyltransferase 1 Targeting Using Guadecitabine Inhibits Prostate Cancer Growth by an Apoptosis-Independent Pathway

**DOI:** 10.3390/cancers15102763

**Published:** 2023-05-15

**Authors:** Dev Karan, Manohar Singh, Seema Dubey, Peter J. Van Veldhuizen, Yogen Saunthararajah

**Affiliations:** 1 Department of Pathology, MCW Cancer Center, Prostate Cancer Center of Excellence, Medical College of Wisconsin, 8701 Watertown Plank Road, Milwaukee, WI 53226, USA; 2 Department of Internal Medicine, University of Rochester Medical Center, Rochester, NY 14642, USA; peter_vanveldhuizen@urmc.rochester.edu; 3 Department of Hematology and Medical Oncology, Cleveland Clinic, Cleveland, OH 44195, USA; saunthy@ccf.org

**Keywords:** prostate cancer, guadecitabine, DNMT1, H3K4 methylation

## Abstract

**Simple Summary:**

We describe the unique role of guadecitabine (gDEC) activating lysine methyltransferases (KMTs) in association with prostate cancer inhibition. This study suggests that one consequence of DNMT1-targeting by gDEC is the upregulation of epigenetic activating enzymes to contribute to the overall prostate cancer phenotype change and anticancer effect independent of apoptosis. Mechanistically, gDEC increases the mono- and di-methylation of H3K4 in prostate cancer cell lines and was associated with DNMT1 depletion. Importantly, DNMT1-targeting with gDEC to reverse aberrant epigenetic repression of tumor suppressor programs suggests the therapeutic potential of treating prostate cancer.

**Abstract:**

Epigenetic alterations such as DNA methylation and histone modifications are implicated in repressing several tumor suppressor genes in prostate cancer progression. In this study, we determined the anti-prostate cancer effect of a small molecule drug guadecitabine (gDEC) that inhibits/depletes the DNA methylation writer DNA methyltransferase 1 (DNMT1). gDEC inhibited prostate cancer cell growth and proliferation in vitro without activating the apoptotic cascade. Molecular studies confirmed DNMT1 depletion and modulated epithelial-mesenchymal transition markers E-cadherin and β-catenin in several prostate cancer cell lines (LNCaP, 22Rv1, and MDA PCa 2b). gDEC treatment also significantly inhibited prostate tumor growth in vivo in mice (22Rv1, MDA PCa 2b, and PC-3 xenografts) without any observed toxicities. gDEC did not impact the expression of androgen receptor (AR) or AR-variant 7 (AR-V7) nor sensitize the prostate cancer cells to the anti-androgen enzalutamide in vitro. In further investigating the mechanism of cytoreduction by gDEC, a PCR array analyses of 84 chromatin modifying enzymes demonstrated upregulation of several lysine-specific methyltransferases (KMTs: KMT2A, KMT2C, KMT2E, KMT2H, KMT5A), confirmed by additional expression analyses in vitro and of harvested xenografts. Moreover, gDEC treatment increased global histone 3 lysine 4 mono-and di-methylation (H3K4me1 and H3K4me2). In sum, gDEC, in addition to directly depleting the corepressor DNMT1, upregulated KMT activating epigenetic enzymes, activating terminal epithelial program activation, and prostate cancer cell cycling exits independent of apoptosis.

## 1. Introduction

Although several anti-androgen and other drug classes are available for treating prostate cancer, the disease inevitably relapses within 2–3 years. Such emergence of castration-resistant prostate cancer (CRPC) causes approximately 30,000 deaths per year in the United States, the second leading cause of cancer-related deaths in men [1,2]. There is hence a need for new treatments that are not cross-resistant with current therapies.

Castration-resistant metastatic prostate cancer accumulates both genetic and epigenetic changes. Epigenetic alterations such as DNA methylation and histone modifications are implicated in the repression of several tumor suppressor genes in prostate cancer [3,4,5], and aberrant methylation patterns in circulation-free DNA in prostate cancer samples can be used to improve prostate cancer diagnosis and prognosis [6]. DNA methylation is written by DNA methylating enzymes DNMT1, DNMT3a, and DNMT3b. Specifically, DMNT1 expression was upregulated in prostate cancer cell lines and tumor tissues compared to non-cancer benign prostatic hyperplasia (BPH)-1 cell lines and BPH tissues [7,8]. The overexpression of DNMT1 is known to promote prostate tumor formation in the transgenic adenocarcinoma of the mouse prostate (TRAMP) model [3,9].

Dysregulation of lysine (K) methyltransferases (MTs) has also been frequently documented in human cancers. Translocations and mutations in KMTs are highly recurrent in hematopoietic malignancies [10,11]. In addition, deregulated KMT expression has been described in prostate cancer [12,13]. Notably, DNA and lysine methylating apparatus are interrelated and interdependent in chromatin modification in vivo [14,15]. However, molecular crosstalk between the DNMTs and KMTs has not been described in prostate cancer studies.

Pharmacological inhibitors/depletors of DNMT1, decitabine (5-aza-2′-deoxycytidine) and 5-azacitidine, are first-generation DNA hypomethylating agents and approved treatments for myeloid malignancies [16,17,18]. However, the short half-lives, uneven tissue distribution, and toxicities at high doses are major limitations [19]. Therefore, there has been a continuous interest in developing new DNMT1-targeting drugs. Guadecitabine (gDEC), also known as SGI-110, is a second-generation agent derived from decitabine. gDEC is a dinucleotide antimetabolite of decitabine linked by a phosphodiester bond to guanosine, and is not recognized by cytidine deaminase that rapidly catabolizes decitabine in vivo. Thus, gDEC application is hoped to increase exposure times for DNMT1-targeting in vivo [20,21]. The effect of gDEC inhibiting DNMT1 has been shown in several cancer types, including pancreatic, ovarian, and hepatocellular carcinoma [19,22,23]. There is no report, however, on the use of gDEC in targeting prostate cancer. We demonstrated that gDEC depletes DNMT1 from prostate cancer cells to inhibit their proliferation without activating apoptosis but with epithelial changes that decrease migration and invasion in vitro and in vivo murine xenograft models. The targeting of DNMT1 also resulted in an increase in histone methylation, drawing a functional link between DNMT1 and histone methyltransferases in prostate cancer.

## 2. Materials and Methods

### 2.1. Cell Culture Maintenance

Human prostate cancer cell lines LNCaP (CRL-1740), 22Rv1 (CRL-2505), MDA PCa 2b (CRL-2422), PC-3 (CRL-1435), and DU145 (HTB-81) were purchased from American Type Culture Collection (ATCC, Manassas, VA, USA) and maintained in the culture media in a humidified incubator with 5% CO_2_ at 37 °C as per instructions. The cell lines were authenticated using short tandem repeat (STR) profiling within the last three years (Labcorp, Burlington, NC, USA). C4-2B (CRL-3315) cells were recently purchased from ATCC. Particularly, MDA PCa 2b cells were maintained in an F-12K culture medium (ATCC, Cat. #30-2004) supplemented with 20% non-heat-inactivated fetal bovine serum (ATCC, Cat. #30-2020), 0.005 mM phosphoethanolamine (Sigma, St. Louis, MO, USA, Cat. #P0503), 100 pg/mL hydrocortisone (Sigma, Cat. #H0135), 10 ng/mL mouse EGF (Corning, Glendale, AZ, USA, Cat. #354010), 45 nM sodium selenite (Sigma, Cat. #9133), 25 ng/mL cholera toxin (Sigma, Cat. #C8052), and 0.005 mg/mL human recombinant insulin (Thermo Fisher, Waltham, MA, USA, Cat. #12585-014). LNCaP, 22Rv1, C4-2B, and MDA PCa 2b cells are androgen receptor (AR)-positive, while PC-3 and DU145 are AR-negative. All experiments were performed with mycoplasma-free cell culture.

### 2.2. RNA Isolation and cDNA Synthesis and qPCR

Total RNA was isolated using TRizol reagent (Sigma, Cat. #T9424). RNA was quantified, and integrity was confirmed based on a 260/280 ratio of 2.0 ± 0.1 using a Biotek multimode reader. The cDNA was synthesized as per standard protocol using 1μg of total RNA, Oligo d(T) primer (Cat. #N8080128), and SuperScript™ III Reverse Transcriptase (Cat. #18080044) from Thermo Fisher. Quantitative polymerase chain reaction (qPCR) was performed in the thermocycler (Applied Biosystems, Waltham, MA, USA) using pre-designed gene-specific primers (Table S1) and PowerUp SYBR-Green master mix reagent (Applied Biosystems, Cat. #A25742), determining the mRNA expression. GAPDH served as a loading control. The fold change was calculated using delta-delta (dd)CT values with reference to control.

### 2.3. RT2 Profiler PCR Array

To analyze a panel of 84 genes of the Human Epigenetic Chromatin Modification Enzymes in prostate cancer, we used an RT2 Profiler™ PCR Array (Qiagen, Frederick, MD, USA, Cat. #PAHS-085ZA). Fold change was calculated using the delta-delta (dd)CT method, and the heat map was generated by Qiagen RT^2^ Profiler PCR Data Analysis online software. MDA PCa 2b cells were treated with guadecitabine (Selleckchem, Houston, TX, USA, Cat. #S7013) at a concentration of 10 μM for 7 days; total RNA was reverse-transcribed to synthesize cDNA as above.

### 2.4. Cell Proliferation and Annexin-V Assay

Cell growth proliferation was assessed by trypan blue (Thermo Fisher, MA, USA, Cat. #15250061) exclusion assay and IncuCyte. Trypan blue dye penetrates dead cells and is distinguished from the live cells by staining in blue color. For each experiment, 5 × 10^4^ cells were seeded in 6-well plates and treated with 10 µM gDEC. The number of live cells between the control and treatment groups was determined using a hemocytometer. The effect of gDEC on cell proliferation and apoptosis (annexin-v) was also monitored using IncuCyte live cell imaging system. PC-3 and DU145 cells (3000 cells per well) were seeded in 96-well plates. Following overnight incubation, the cells were treated with 2, 5, and 10 µM gDEC in media containing IncuCyte annexin-v dye as per instructions (Sartorius, New York, NY, USA).

### 2.5. Colony Formation Assay

LNCaP, 22Rv1, MDA PCa 2b, PC-3, and DU145 cells were seeded in 6-well plates (2000 cells/well) and incubated overnight at 37 °C. The next day, cells were treated with guadecitabine at a given concentration of 10 µM for 4 days, and the cells were replenished with fresh medium without gDEC. After 10 days, the cells were washed with phosphate buffer saline (PBS) and fixed with ice-cold methanol at −20 °C for 10 min, followed by overnight staining with QC Colloidal Coomassie Stain (BioRad, Hercules, CA, USA, Cat. #1610803) at room temperature. The plates were washed with deionized water, and pictures were taken.

### 2.6. Invasion Assay

The cell invasion assay was performed using a 24 well trans-well invasion chamber with an 8.0 µm PET Membrane (Corning, Glendale, AZ, USA, Cat. #354480). Briefly, the invasion chamber was removed from −20 °C and allowed to come to room temperature. The invasion chamber was rehydrated in serum-free media for 2 h at 37 °C in a tissue culture incubator. The media (500 µL) containing 10% FBS was added to the lower compartment of the trans-well as a chemo-attractant. Cells were pre-treated with 10 μM gDEC for 3 Days. After 3 days, 50,000 cells were suspended in 500 µL of the media containing 1% FBS, 10 μM gDEC, and added to the upper compartment of the invasion chamber. The same number of cells without gDEC treatment served as a control. Following the incubation for 48 h at 37 °C in the tissue culture incubator, cells were fixed with ice-cold methanol at −20 °C for 10 min and stained with Coomassie brilliant blue. The remaining cells from the upper compartment were removed using cotton-tipped swabs with gentle swabbing.

### 2.7. RNA Interference

Small interfering RNA (siRNA) targeting KMT2A, KMT2C, and DNMT1 were purchased from Origene (Rockville, MD, USA). Xfect transfection reagent kit was obtained from Takara Bio USA Inc. (San Jose, CA, USA), and the siRNA transfection reactions were performed according to the manufacturer’s protocol.

### 2.8. Western Blot Analysis

Protein cell lysates were prepared using radioimmunoprecipitation assay (RIPA) buffer (Cat. #R0278) supplemented with protease and phosphatase inhibitors cocktail (Cat. #P2850) from Sigma-Aldrich (St. Louis, MO, USA). Protein concentration was measured by Bradford assay. Protein sample (20–30 µg) was mixed with 4× laemmli buffer (Bio-Rad, Hercules, CA, USA, Cat. #1610747) and resolved on the SDS-PAGE, blotted on PVDF membrane (Millipore, MA, USA, Cat. #IPVH00010). The membrane was blocked in 5% bovine serum albumin (BSA: Cat. #A9647; Sigma) in 1× TBS containing 0.01% tween-20 and then incubated overnight at 4 °C with the following primary antibodies: DNMT1 (Cat. #Ab13537) and anti-histone H3 (Cat. #Ab1791) from Abcam (Cambridge, UK), E-cadherin (Cat. #3195), β-catenin (Cat. #8480), PARP (Cat. #9542), cleaved caspase3 (Cat. #9661), KMT2A (Cat. #14197S), GAPDH (Cat. #5174) from Cell Signaling Technology, Inc (Danvers, MA, USA), and H3K4me1 (Cat. #710795), H3K4me2 (Cat. #710796), and H3K4me3 (Cat. #39159) from Thermo Fisher (Waltham, MA, USA). Following the primary antibody incubation, the blots were washed and incubated with HRP-conjugated with respective secondary antibodies. Specific protein bands were visualized by the chemiluminescence system (Cat. #NEL122001EA, Perkin Elmer, Waltham, MA, USA) on the Invitrogen iBright FL1000 imaging system. GAPDH (1:8000) was used as a loading control.

### 2.9. Tumor Xenograft Model

Animal experiments were performed as per the approved study protocol by the institutional animal care and use committee (IACUC) at the Medical College of Wisconsin. Six-to-seven-week-old male athymic nude mice were purchased from the National Cancer Institute. Tumors were established by subcutaneous inoculation of 22Rv1 (2 × 10^6^), PC-3 (2 × 10^6^), or MDA PCa 2b (4 × 10^6^) cells into the right flank of the mice. After visible tumor development, mice were randomized into a vehicle (control) and gDEC treatment groups. Guadecitabine was resuspended in sterile phosphate buffer saline (1 × PBS) and administrated by intraperitoneal injection of 100 μg/animal (3.3 mg/kg body weight) twice a week for 3 weeks in 22Rv1 and MDA PCa 2b tumor cell bearing mice, and 50 μg/animal (~1.65 mg/kg body weight) thrice a week for 3 weeks in PC-3 xenograft animal. The control group of mice was injected with 1 × PBS. Tumor size was measured, and the tumor volume was calculated as described previously [24].

### 2.10. Statistical Analysis

All the statistical analysis was performed using statistical software (GraphPad Prism9). Significant differences between various groups were analyzed by the analysis of variance (ANOVA) and nonparametric Mann–Whitney test.

## 3. Results

### 3.1. DNMT1 Targeting with Guadecitabine (gDEC) Inhibited Prostate Cancer Cell Growth

**Figure 1:**
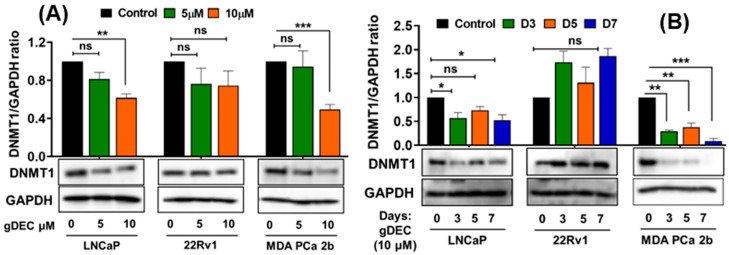
Guadecitabine inhibits DNMT1 expression in prostate cancer cell lines. LNCaP, 22Rv1, and MDA PCa 2b cells were treated with (**A**) 5 and 10 µM of gDEC for 5 days and (**B**) 10 µM gDEC for 3, 5, and 7 days and DNMT 1 protein expression was determined by western blot assay. GAPDH was used as a loading control. The data are represented by the mean ± SEM of three independent sets of experiments. Significance levels are * *p* < 0.05, ** *p* < 0.01, *** *p* < 0.001, and ns = non-significant. Original western blots are presented in File S1.

**Figure 2:**
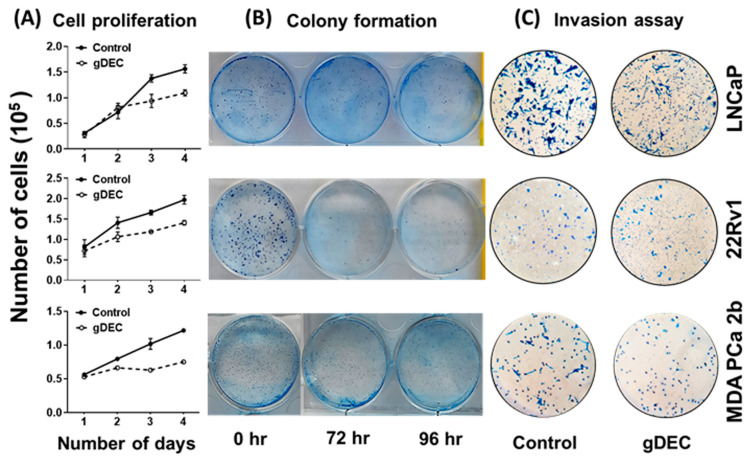
Anticancer effect of gDEC targeting prostate cancer. (**A**) Effect of gDEC on prostate cancer cell proliferation. LNCaP, 22Rv1, and MDA PCa 2b cells were treated with 10 μM gDEC, and viable cells were counted by trypan blue assay. Data were represented by mean ± SEM of three independent experiments. (**B**) The relative cell survival was analyzed by colony formation assay, and (**C**) inhibition of cell invasion was analyzed by Matrigel invasion assay.

To identify an effective dose of gDEC for targeting prostate cancer, we performed an MTT assay on 22Rv1 cells using a wide range of gDEC concentrations (0.625 to 25 µM) for 24, 48, and 72 h. However, an IC50 value appeared beyond the tested gDEC concentrations (Figure S1A). Next, we examined the effect of gDEC (2, 5, and 10 µM) on inhibiting DNMT1. In 22Rv1 cells, gDEC effect at 2, 5, or 10 µM in 3 day treatment remained feeble in suppressing DNMT1. However, gDEC at 10 µM showed a substantial decrease in DNMT1 level in DU145 cells (Figure S1B). Furthermore, we confirmed that gDEC significantly decreased DNMT1 protein levels in prostate cancer cell lines in a dose- and time-dependent manner (Figure 1). gDEC inhibited cell growth, and colony formation and invasion in a panel of prostate cancer cell lines: LNCaP, 22Rv1, and MDA PCa 2b (Figure 2). Similar observations of gDEC-directed inhibition of DNMT1, cell growth, and colony formation were validated in PC-3 and DU145 cell lines (Figure S2). Interestingly, we found that gDEC inhibited prostate cancer cell growth and proliferation in vitro without activating the apoptotic cascade as measured by apoptotic markers poly (ADP-ribose) polymerase (PARP) and caspase-3 cleavage (Figure S3).

**Figure 3:**
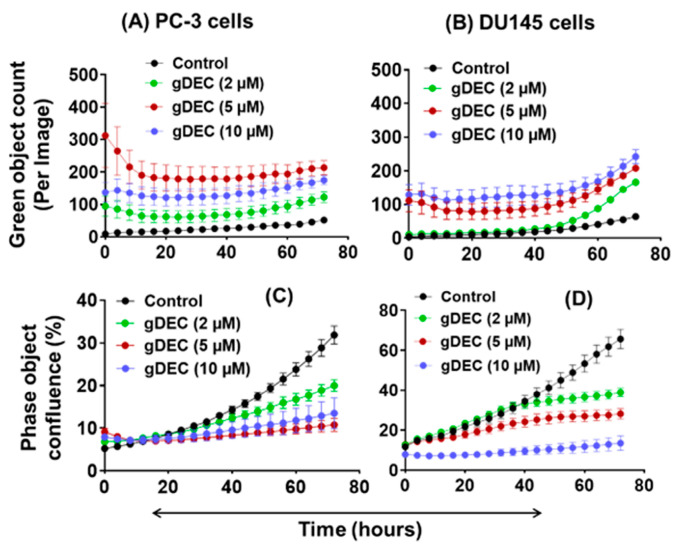
IncuCyte-monitored real time analysis of gDEC effect on green object count (annexin-v: (**A**,**B**)) and cell growth (phase object confluence: (**C**,**D**)) in PC-3 and DU145 cells. To analyze the gDEC effect, 3 × 10^3^ cells were seeded per well in 96-well plates and the image counts were captured every 4 h for 72 h.

To further explore the effect of apoptotic induction, we performed IncuCyte analysis for green object count (annexin-v) in real time. A nonparametric Mann–Whitney test showed no significant differences between the 5 µM and 10 µM of gDEC at any time point (Figure 3A,B). However, gDEC at 2 µM showed an increase in annexin-v positive DU145 cells at 72 h (*p* = 0.028) compared to 24 h. Furthermore, the green object count (annexin-v) at 72 h remains almost same at any given gDEC concentration. The phase object confluence analysis showed a dose-dependent cell growth attenuation corresponds to trypan blue assay for cell proliferation (Figure 3C,D). Together with the lack of caspase-3 and PARP cleavage, these observations indicated that induction of apoptosis may not be a desirable course of gDEC action in suppressing prostate cancer cell growth.

**Figure 4:**
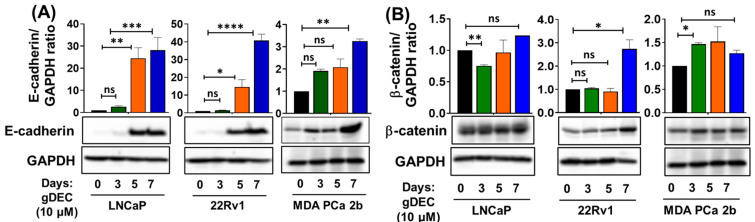
Time-dependent effect of gDEC on the regulation of EMT markers. Protein expression of (**A**) E-cadherin and (**B**) β-catenin in LNCaP, 22Rv1, and MDA PCa 2b cell lines. GAPDH served as a loading control. A total of 2 × 10^5^ cells were plated in six-well plates and were treated with 10 µM gDEC. Cells were harvested at the given time-point of days 3, 5, and 7 to prepare protein lysates for western blot analysis. The bar graph represents E-cadherin or β-catenin/GAPDH ratio represented by mean ± SEM. Significance levels are * *p* < 0.05, ** *p* < 0.01, *** *p* < 0.001, **** *p* < 0.0001, and ns = non-significant. Original western blots are presented in File S1.

To better understand the pathway by which gDEC inhibited prostate cancer cell growth/invasion, we examined expression levels of epithelial-mesenchymal transition markers E-cadherin and β-catenin. gDEC significantly increased E-cadherin protein levels in all three cell lines, while the β-catenin protein expression remained inconsistent (Figure 4).

### 3.2. gDEC Inhibits Prostate Cancer Cell Growth In Vivo Xenograft Models

To further evaluate the therapeutic efficacy of gDEC, tumor-bearing mice were treated with intraperitoneal injections of gDEC for 3 weeks. Mice harboring tumors with 22Rv1 or MDA PCa 2b received 100 μg (3.3 mg/kg body weight) of gDEC/animal twice a week, while the mice bearing PC-3 tumors received 50 μg (1.65 mg/kg body weight) of gDEC per animal thrice a week.

**Figure 5:**
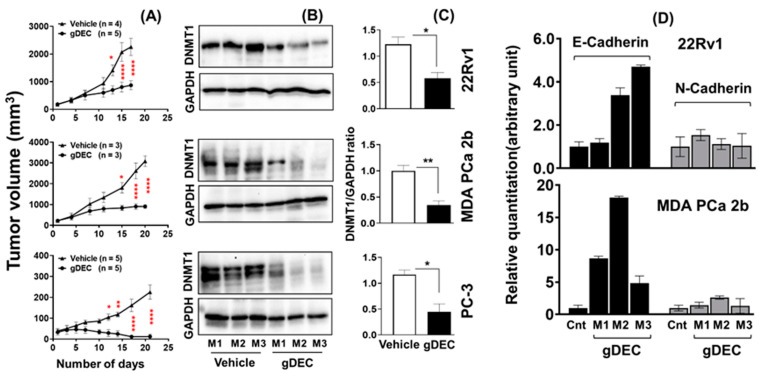
Therapeutic efficacy of gDEC in targeting prostate cancer in vivo. Male mice were inoculated subcutaneously with 22Rv1 (2 × 10^6^), PC-3 (2 × 10^6^), or MDA PCa 2b (4 × 10^6^) cells into the right flank. (**A**) Inhibition of 22Rv1, MDA PCa 2b, and PC-3 cells tumor growth in athymic male nude mice following gDEC treatment. (**B**) Excised tumors were analyzed for DNMT1 protein expression from three individual mice (M1, M2, and M3) for each cell type xenograft. (**C**) The bar graph represents the DNMT1 intensity compared to GAPDH from three animals per cell type xenografts. (**D**) E-cadherin and N-cadherin mRNA expression in tumor xenografts. Error bar represents mean ± SEM. Significant levels are * *p* < 0.05, ** *p* < 0.01, and **** *p* < 0.0001. Original western blots are presented in File S1.

Three days after the last gDEC injection, mice were sacrificed to excise tumors for the mRNA and protein analysis. gDEC treatment significantly inhibited tumor growth (*p* < 0.0001) compared to the vehicle-treated control group of mice in all three cell line models (Figure 5A). We confirmed that gDEC produced the intended molecular pharmacodynamic effect of DNMT1 depletion in the tumors (Figure 5B,C). gDEC-treated mice also showed an increased level of E-cadherin mRNA in tumor xenograft while N-cadherin expression remains unchanged (Figure 5D). There was no visible effect of gDEC on the animal behavior, including drooling and hunching, and change in body weight (22Rv1: *p* = 0.763, PC-3: *p* = 0.242) at two different doses (Figure S4).

### 3.3. Effect of gDEC on AR Regulation and Chemo-Sensitization of Prostate Cancer Cells

**Figure 6:**
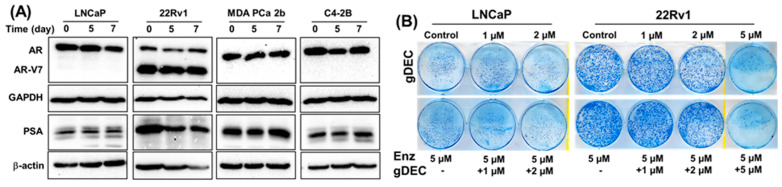
Effect of gDEC on AR and PSA regulation and chemo-sensitization. (**A**) Representative blots of AR and PSA protein expression in a panel of prostate cancer cell lines LNCaP, 22Rv1, MDA PCa 2b, and C4-2B treated with 10 µM gDEC for 5 and 7 days. GAPDH and β-actin served as a loading control. (**B**) Representative pictures of colony formation. LNCaP (6000 cells/well) and 22Rv1 (2000 cells/well) cells were seeded in 6-well plates. Following 24 h, the cells were treated with 5 µM enzalutamide, 1 μM, 2 μM, or 5 μM gDEC as a single agent and in combinations. After 72 h of treatment, the media was removed, and the cells were allowed to grow for 1 week in complete media to perform a colony formation assay. These data were repeated twice with similar results. Original western blots are presented in File S1.

Anti-androgen therapy is the mainstay therapy for prostate cancer; therefore, we examined if the gDEC could sensitize prostate cancer cells to the anti-androgen drug enzalutamide and AR-regulated prostate-specific antigen levels (PSA). Protein expression analysis of AR and PSA in AR-expressing cells revealed no effect of gDEC on AR, its enzalutamide-resistant variant (AR-V7), or PSA (Figure 6A). Furthermore, gDEC and enzalutamide combinations of non-cytotoxic doses did not show an additive or synergistic effect in an in vitro colony formation assay (Figure 6B). It seems that gDEC is ineffective at 1 and 2 μM, and there is no synergistic or additive effect of gDEC and enzalutamide combination in vitro. While there is a reduction in colony formation at 2 μM, apparently, gDEC at 5 μM dramatically reduced the colony formation in 22Rv1 cells. However, the combination with enzalutamide remains ineffective, indicating that gDEC alone is a potent inhibitor of cell survival and proliferation.

### 3.4. gDEC Pathway of Action against Prostate Cancer

**Figure 7:**
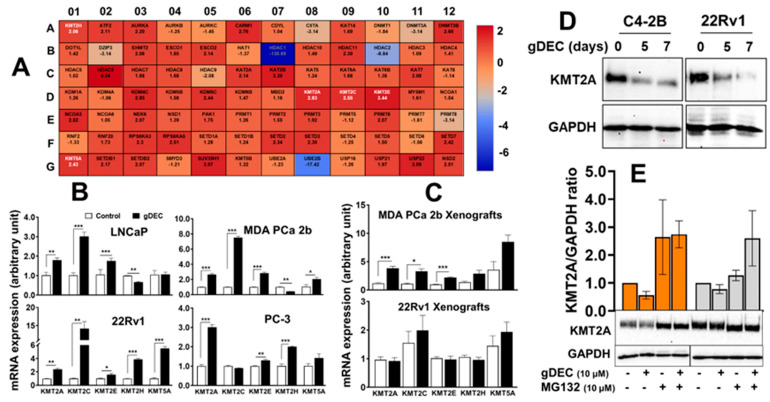
Differential expression analysis of human epigenetic chromatin modification enzymes following gDEC treatment using RT^2^ Profiler PCR Array. (**A**) The heat map showed differential expression of genes in MDA PCa 2b cells, and each block represents fold changed (log2) values upregulated (red color) or downregulation (blue color) compared to the untreated control. (**B**) Validation of mRNA expression of the selected differentially expressed KMTs (KMT2A, KMT2C, KMT2E, KMT2H, and KMT5A) in 4 different cell lines (LNCaP, 22Rv1, MDA PCa 2b, and PC-3) in vitro following the treatment with 10 μM gDEC for 5 days. (**C**) Ex vivo tumor xenografts of MDA PCa 2b and 22Rv1 cells. (**D**,**E**) gDEC results decrease in KMT2A protein, which is rescued following treatment with proteasomal inhibitor (MG132). Data was represented by mean ± SEM, * *p* < 0.05, ** *p* < 0.01, *** *p* < 0.001. Non-significant values are not provided. Original western blots are presented in File S1.

In further investigating the molecular mechanism by gDEC, unbiased analysis of 84 human epigenetic chromatin modification enzymes revealed a differential expression of 37 genes compared to vehicle control (Figure 7A). Among the upregulated genes, we observed a significant increase in lysine-specific methyltransferases (KMTs), including KMT2A (2.83-fold), KMT2C (2.55 fold), KMT2E (3.44-fold), KMT2H (2.06 fold), and KMT5A (2.43 fold) in gDEC-treated cells, and validated the differential expression at the mRNA level in vitro cell culture and xenograft tumors (Figure 7B,C). The identified KMTs profile from the qPCR array was consistent with in vitro gDEC-treated 22Rv1 cells; however, it was not comparable in 22Rv1 xenograft tumors, even though gDEC treatment significantly inhibited 22Rv1 tumor growth in mice. Validation at the protein level showed that KMT2A expression was reduced following gDEC treatment (Figure 7D). Further analysis revealed that KMT2A protein undergoes proteasomal degradation as the use of proteasome inhibitor (MG132) rescued KMT2A protein (Figure 7E), supporting the previous report that E3 ligases mediate KMT2A protein degradation [25].

### 3.5. gDEC Regulates H3K4 Methylation Expression in Prostate Cancer

**Figure 8:**
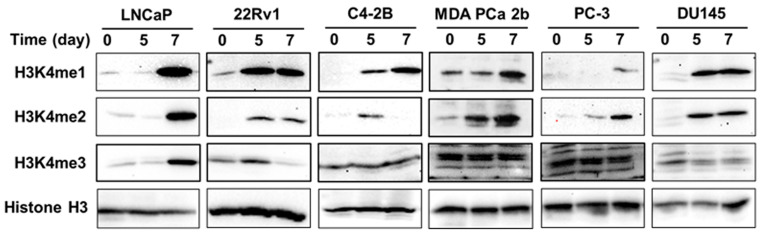
Effect of gDEC on H3K4 methylation in a panel of prostate cancer cell lines (LNCaP, 22Rv1, C4-2B, MDA PCa 2b, PC-3, and DU145) treated with 10 μM gDEC for 5 and 7 days. The protein expression level of H3K4me1, H3K4me2, and H3K4me3 was analyzed by western blot along with GAPDH as a loading control. Original western blots are presented in File S1.

KMTs are known to catalyze H3K4 methylation. Therefore, in association with KMTs, we analyzed the effect of gDEC on regulating H3K4 histone methylation [26]. We observed a consistent increase in the protein level of H3K4me1 and H3K4me2 in a panel of prostate cancer cell lines (LNCaP, 22Rv1, MDA PCa 2b, C4-2B, PC-3, and DU145). Only LNCaP cells showed increased H3K4me3 following gDEC treatment (Figure 8).

**Figure 9:**
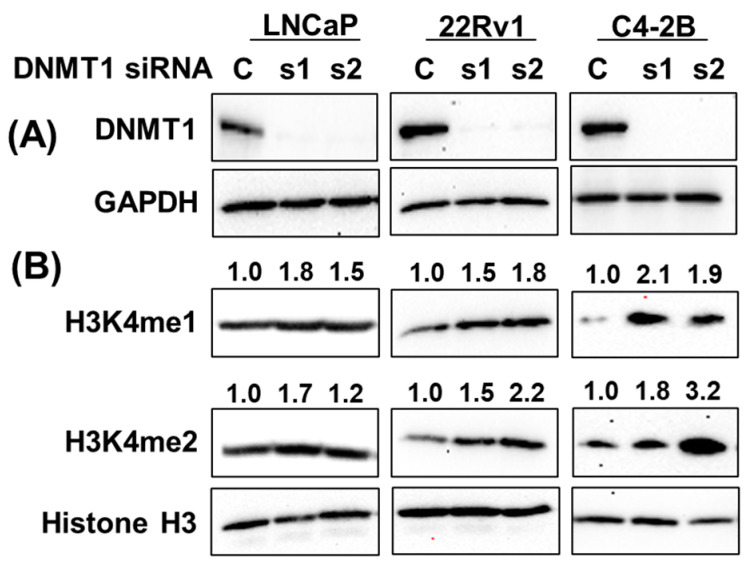
DNMT1-directed mechanistic regulation of H3K4 methylation. Knockdown of DNMT1 using DNMT1-specific siRNA in a panel of prostate cancer cell lines (**A**), and its effect on H3K4me1 and H3K4me2 expression (**B**). Numbers represent the fold change expression of H3K4me1 and H3K4me2 following DNMT1 knockdown with respect to Histone H3. GAPDH served as a loading control. C: siRNA scramble control, while s1 and s2 are two different siRNAs of DNMT1. Original western blots are presented in File S1.

To examine the role of KMTs in regulating H3K4 methylation in prostate cancer, small interfering RNA (siRNA)-directed KMT2A knockdown showed an association with a decrease in H3K4me1 and H3K4me2 in C4-2B and DU145 cells, while it increased in 22Rv1 cells. On the other hand, KMT2C knockdown revealed an increase in H3K4me1 and H3K4me2 in C4-2B, DU145, and 22Rv1 cells, indicating cell-line-specific differences in the regulation of H3K4 methylation (Supplementary Figure S5). On the other hand, siRNA-directed DNMT1 knockdown was directly associated with H3K4 methylation status (Figure 9). DNMT1 knockdown upregulated H3K4me1 and H3K4me2, validating the effect of gDEC in the depletion of DNMT1 and upregulation of H3K4me1 and H3K4me2 in prostate cancer.

## 4. Discussion

Several drugs targeting epigenetic enzymes (‘epidrugs’) have been approved for oncology indications, including inhibitors for DNA methyltransferase (DNMT1), histone deacetylase (HDAC), isocitrate dehydrogenase (IDH), and EZH2 [27]. Among these, the longest clinical experience is with decitabine and azacitidine to target DNMT1. However, low bioavailability and cytotoxicity at high doses are the major limitations of these agents. gDEC is a next-generation DNMT1-targeting drug that has been evaluated in multiple clinical trials in acute myeloid leukemia (AML), lung cancer, and ovarian cancer. It is predicted that gDEC acts similarly to decitabine inhibiting DNMT1. gDEC is incorporated into DNA during the replication process and inhibits DNA methyltransferase activity [19].

Here, for the first time, we demonstrated the anti-prostate cancer effect of gDEC, in several different prostate cancer models, both in vitro and in vivo. We showed that gDEC did not produce major activation of the apoptosis pathway in prostate cancer cells but impedes cell proliferation and invasion, accompanied by a significant increase in the expression of epithelial marker E-cadherin. The promoter region of the E-cadherin gene is methylated in high-grade prostate tumors and cell lines, and treatment with the DNMT1-targeting decitabine restored E-cadherin mRNA expression in prostate cancer cell lines [28]. Previous studies in ovarian cancer also found an increase in E-cadherin protein with DNMT1-targeting therapy, corresponding to a decrease in methylation levels observed at the E-cadherin gene promoter [29]. Several prior studies identified E-cadherin as a tumor suppressor, and it is possible that E-cadherin upregulation played a role in the observed inhibition of prostate cancer growth following gDEC treatment.

One area of translation research is to use epigenetic modifying drugs to potentially re-sensitize cancer cells to standard treatments. For example, gDEC sensitizes platinum-resistant ovarian cancer cells to cisplatin and hepatocellular carcinoma cells to oxaliplatin in vitro and in vivo, via demethylation and re-expression of tumor suppressor genes with subsequent inhibition of tumor growth in mice [23,30]. Similarly, the pretreatment of azacitidine and DNMT1 depletion sensitized metastatic castration-resistant prostate cancer cells to the cytotoxic chemotherapeutics cabazitaxel and docetaxel [31,32]. A recent study showed that decitabine re-establishes the response to enzalutamide in enzalutamide-resistant prostate cancer cell lines with a significant decrease in DNMTs and AR-V7 [33]. However, we did not notice any change in the AR, AR-V7, and AR-regulated PSA protein expression upon gDEC treatment, and gDEC did not facilitate enzalutamide activity in an in vitro assay. In addition, the 22Rv1 cells represent the enzalutamide-resistance stage of prostate cancer. Therefore, the lack of gDEC effect on DNMT1 depletion could be due to therapy-acquired resistance and may require a longer duration of in vitro treatment. Nevertheless, significant inhibition of 22Rv1 tumor xenografts following gDEC treatment aligned with decreased DNMT1.

KMT2 members are known to control the regulation of mono-, di-, and tri-methylation of H3K4, epigenetic marks that mediate gene activation (‘on’ marks), and translocations of KMT2 members that delete the methyltransferase domain are recurrent events in oncogenesis. A low level of H3K4me2 was correlated with poor outcomes in HER-2-positive breast cancer, basal cell carcinomas, and pancreatic adenocarcinomas [34,35]. Likewise, a correlative analysis with the biochemical recurrence of prostate cancer revealed the downregulation of H3K4 methylation in prostate tumor tissues in more than 60% of patients [36]. Interestingly, a meta-analysis of 1474 patients in 10 studies suggested that decreased H3K4me1 methylation might be responsible for poor prognosis in prostate cancer [37]. On the contrary, an upregulation of di- and tri-methylation of H3K4 was coupled with significant tumor growth inhibition in prostate-specific PTEN knockout mice [38]. We demonstrated that gDEC consistently upregulated the level of H3K4me1 and H3K4me2 in a panel of six different prostate cancer cell lines. It is interesting to note that the induction of H3K4 methylation happens around 5–7 days. One plausible explanation for such delayed activation could be the low level of histone methyltransferase enzymes responsible for H3K4 methylation since KMT2A undergoes proteasomal degradation following gDEC treatment. H3K4me1 is a well-established feature of enhancers and promoters in regulating gene expression, where KMT2C provokes H3K4me1 in promoter regions, and KMT2A predominantly regulates both mono- and di-methylation [39,40]. Mechanistically, we observed that KMT2A and KMT2C differentially regulate the H3K4me1 and H3K4me2 in prostate cancer. KMT2A knockdown results in a selective decrease in H3K4me1 and H3K4me2 in two of the tested cell lines, while KMT2C increases H3K4 methylation. Furthermore, we confirmed that H3K4me1 and H3K4me2 were impacted by DNMT1-depletion via gDEC, and we observed a consistent increase in H3K4me1 and H3K4me2 concurrent with growth inhibition in all three prostate cancer models evaluated.

## 5. Conclusions

In sum, we found anti-prostate cancer activity of gDEC that did not appear to be mediated via apoptosis but instead via upregulation of key epithelial-differentiation genes. Our study suggested that one consequence of DNMT1 targeting by gDEC is the upregulation of KMTs, which could mediate the activation of tumor-suppressing programs and contribute to the overall prostate cancer phenotype change and anticancer effect. Overall, these observations support the role of DNMT1-targeting with gDEC to reverse aberrant epigenetic repression of tumor suppressor programs and thereby treat prostate cancer.

## Data Availability

All data to support the proposed study is included in this publication.

## Supplementary Materials

The following supporting information can be downloaded at: https://www.mdpi.com/article/10.3390/cancers15102763/s1, File S1: Original western blots. Figure S1: (A) MTT-based cell viability analysis in 22Rv1 prostate cancer cells treated with 0.625 to 25 µM of gDEC. DNMT1 inhibition, (B) dose titration analysis of gDEC (2, 5, and 10 µM) following 3-day treatment of 22Rv1 and DU145 cells inhibiting DNMT1. Figure S2: effect of gDEC on PC-3 and DU145 prostate cancer cell lines. (A) DNMT1 inhibition, (B) anti-cell proliferation, and (C) inhibition of colony formation. Experimental details are the same as detailed in Figure 1 and Figure 2. Significant levels are *** *p* < 0.001, **** *p* < 0.0001. Figure S3: Effect of gDEC on apoptosis markers in prostate cancer cells. (A) LNCaP, 22Rv1, MDA PCa 2b, and PC-3 cells were treated with 10 µM gDEC for 3 and 5 days, and the protein samples were analyzed for cleaved PARP and caspase-3 expression with GAPDH as a loading control. (B) Re-examination of 22Rv1 cells for PARP and caspase-3 cleavage with positive controls validating the effect of gDEC. Figure S4: Effect of gDEC on (A) tumor weight in 22Rv1 and MDA PCa 2b xenografts. On day 23, the tumor was excised and weighed. (B) Body weight in mice challenged with 22Rv1 and PC-3 cells compared to vehicle controls. There was no significant change (22Rv1: *p* = 0.763 and PC-3: *p* = 0.242) in the body weight between control and gDEC-treated animals. Error bar represents mean ± SEM. Significant level is ** *p* < 0.01 for tumor weight. Figure S5: (A) analysis of siRNA-directed KMT2A and KMT2C knockdown, and (B) effect of KMT2A and KMT2C knockdown on H3K4 methylation. C represents a siRNA scramble control, while s1 and s2 are two different siRNAs for KMT2A and KMT2C. This experiment was repeated 2–3 times from independent cell preparations with similar results. Table S1: list of pre-designed SYBR GREEN primers sequence from Sigma used for RT-qPCR.
