# Supplementary material for: DNA Methyltransferase 1 Targeting Using Guadecitabine Inhibits Prostate Cancer Growth by an Apoptosis-Independent Pathway
Source: Cancers (Basel). 2023 May 15;15(10):2763. doi: 10.3390/cancers15102763 (PMC10216613; doi:10.3390/cancers15102763)

Representative blots of Figure 1

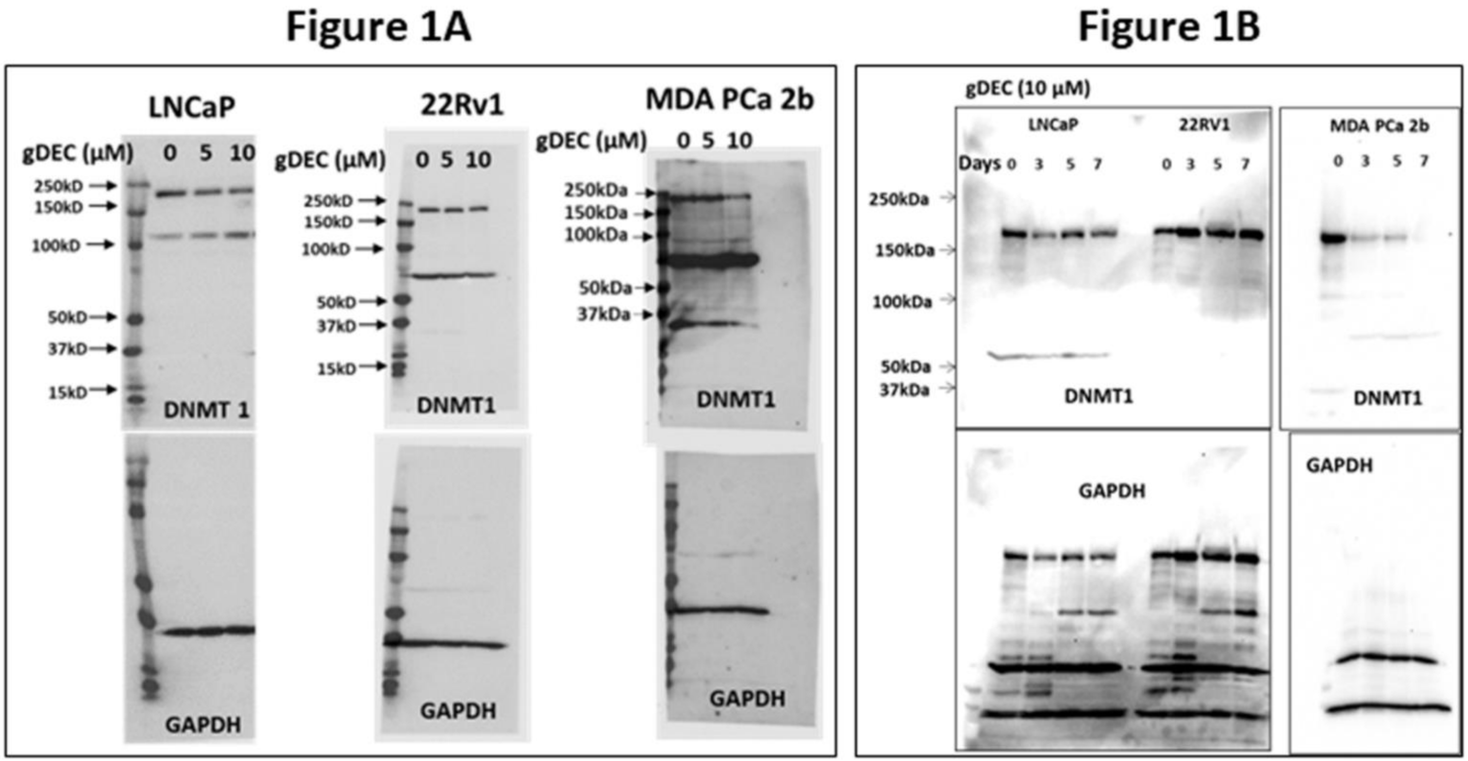

Representative blots of Figure 4

Figure 4A

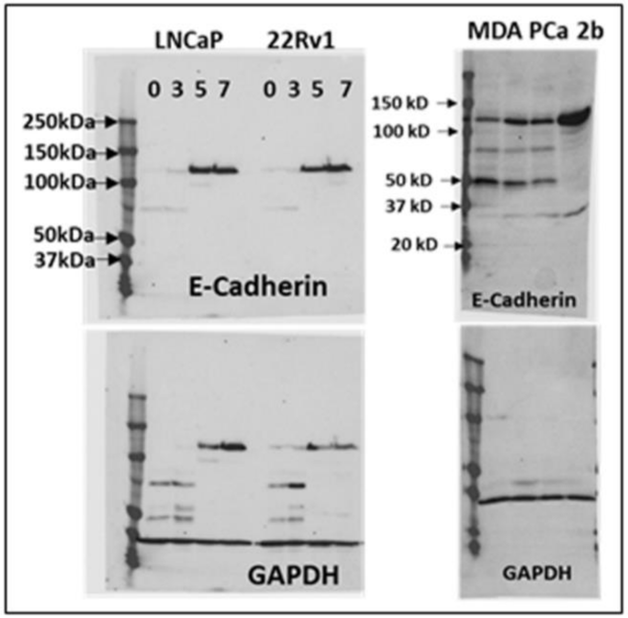

Figure 4B

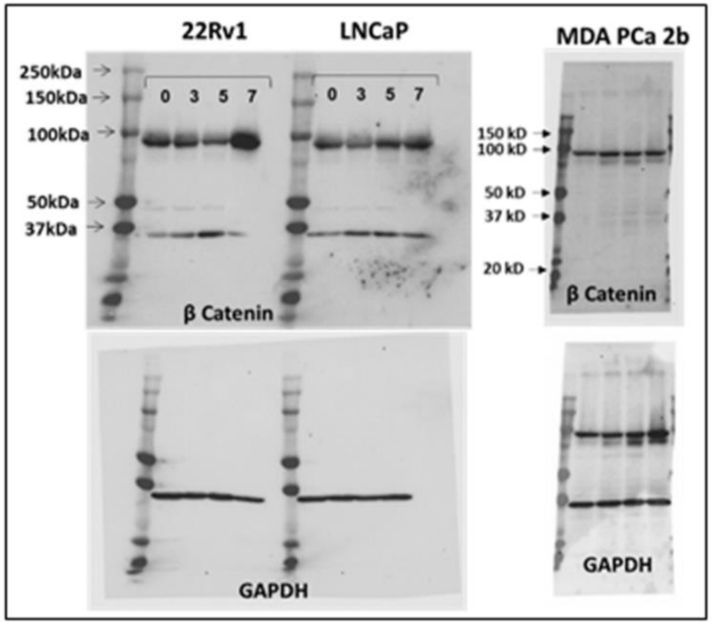

Representative blots of Figure 5B

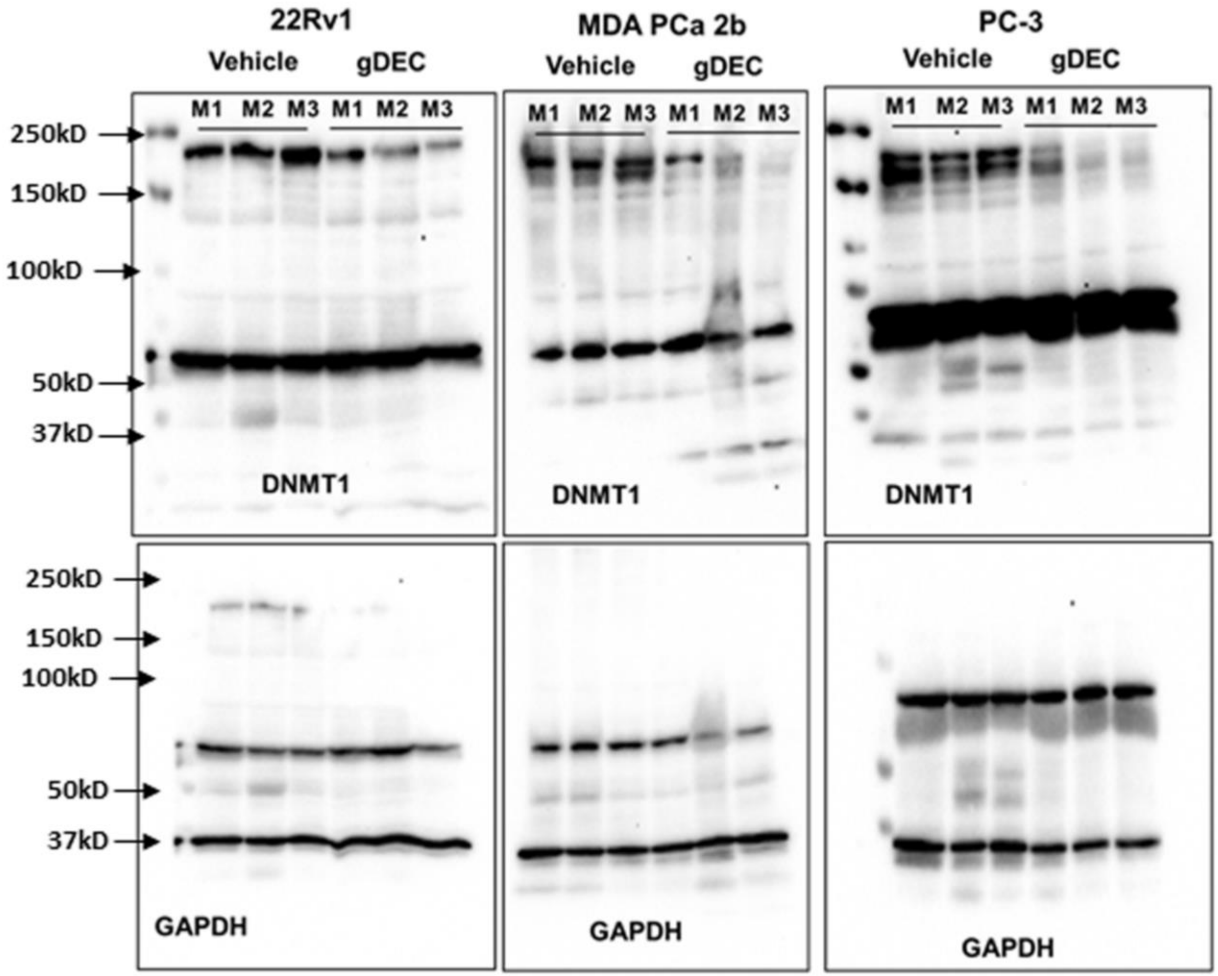

Representative blots of Figure 6A

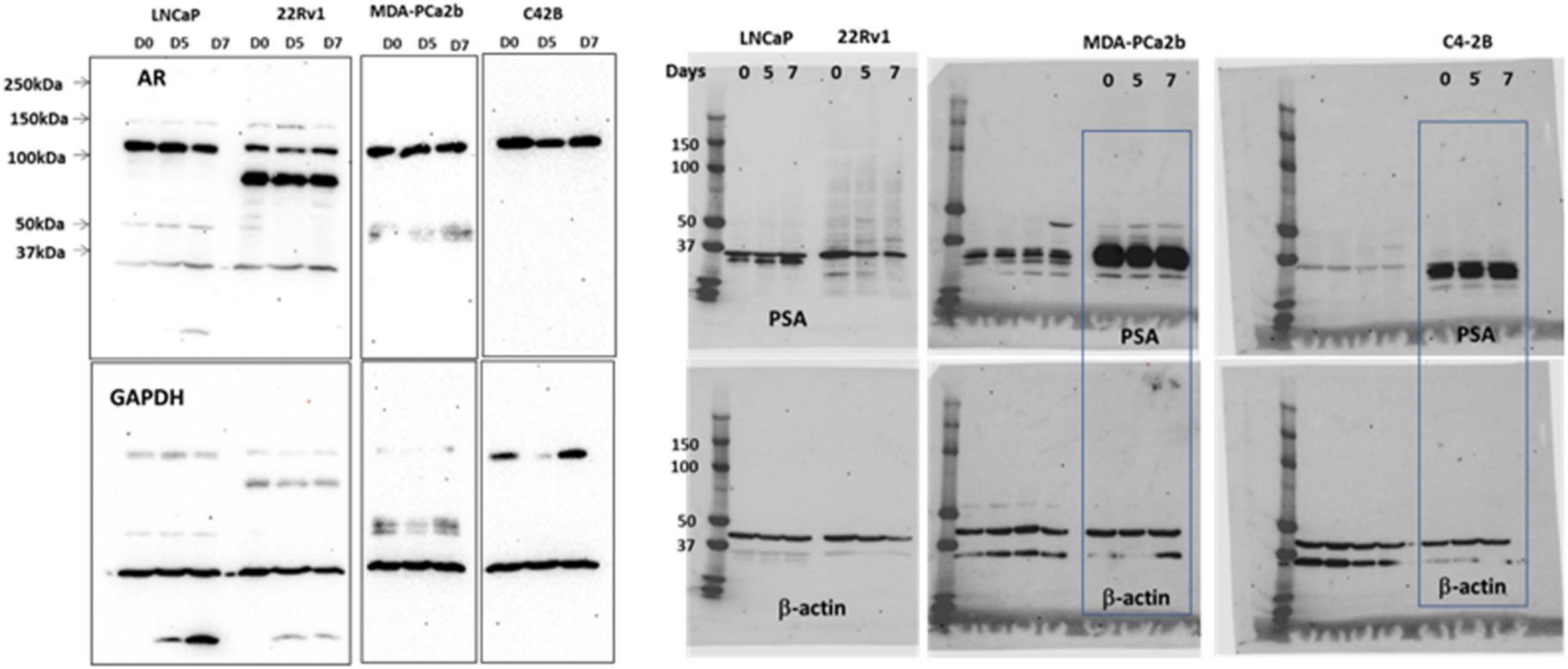

Representative blots of Figure 7

Figure 7D

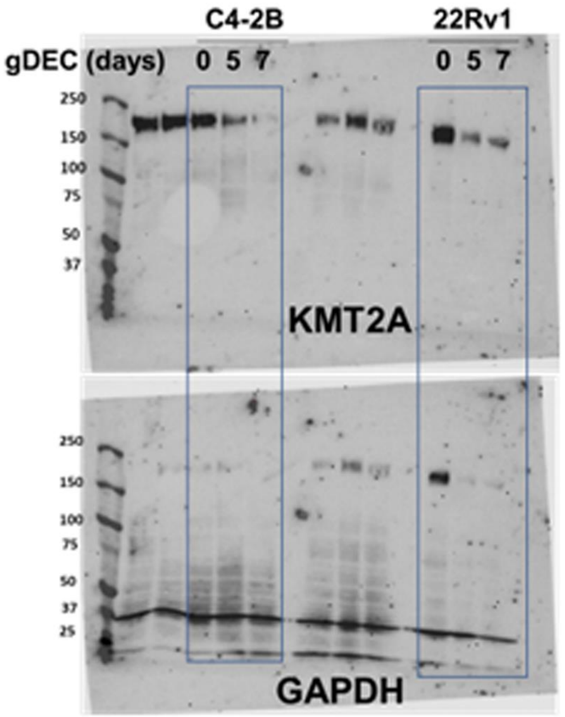

Figure 7E

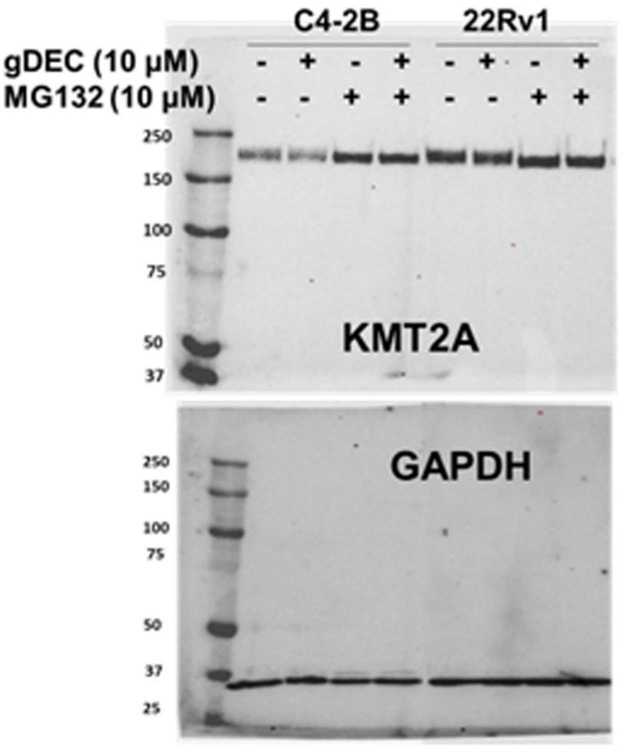

Representative blots of Figure 8

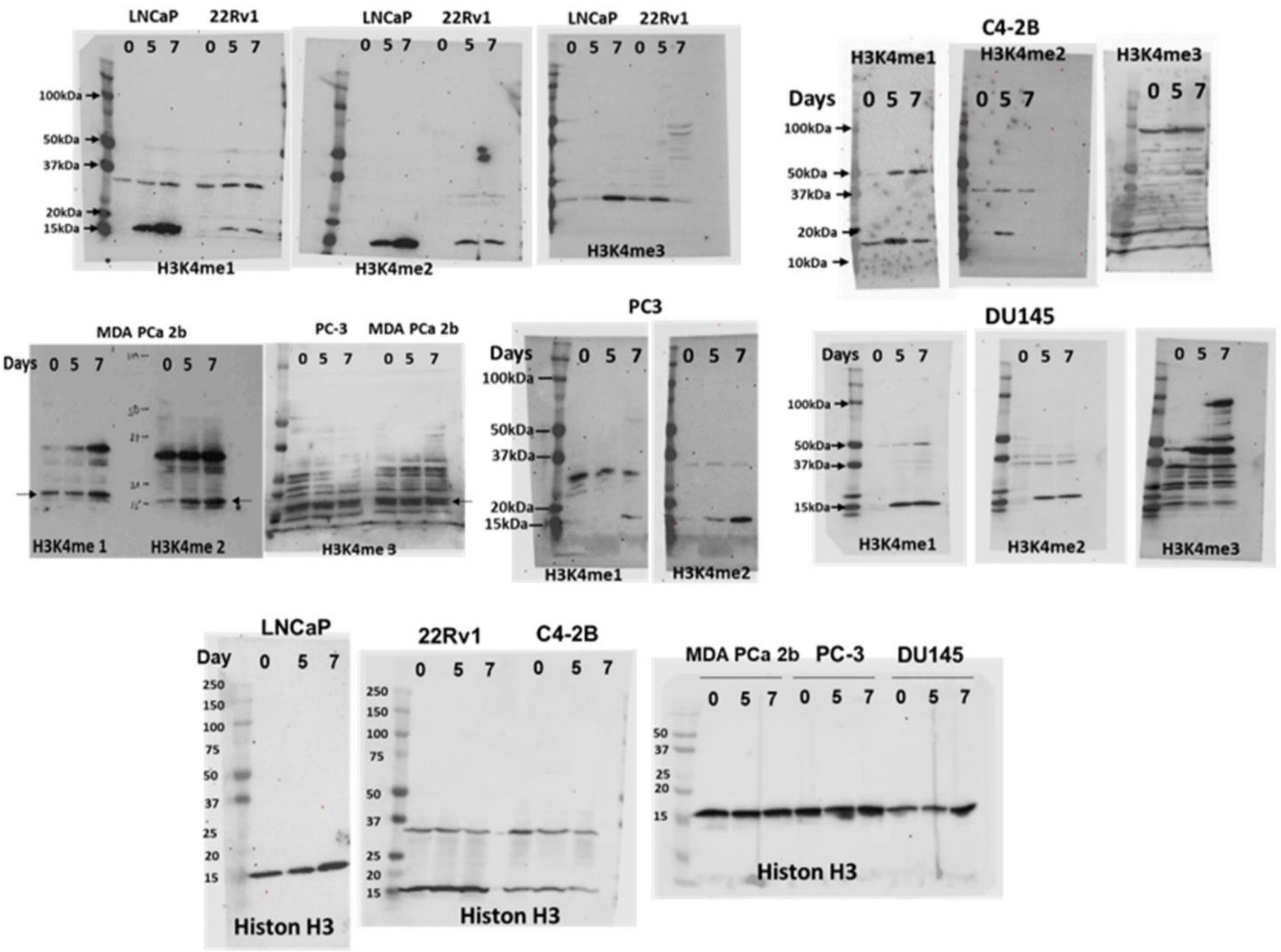

Representative blots of Figure 9

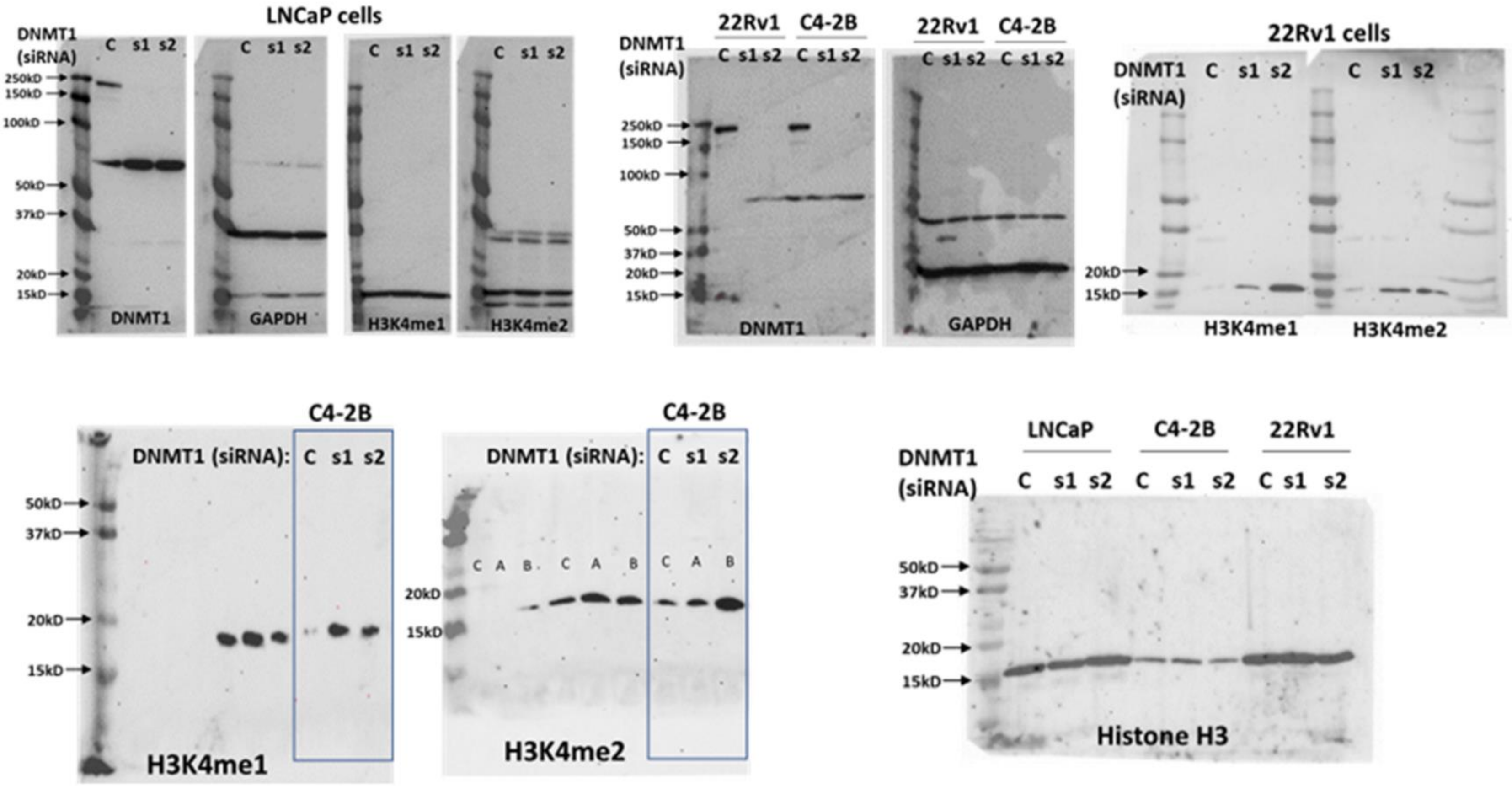

Representative blots: SF1B

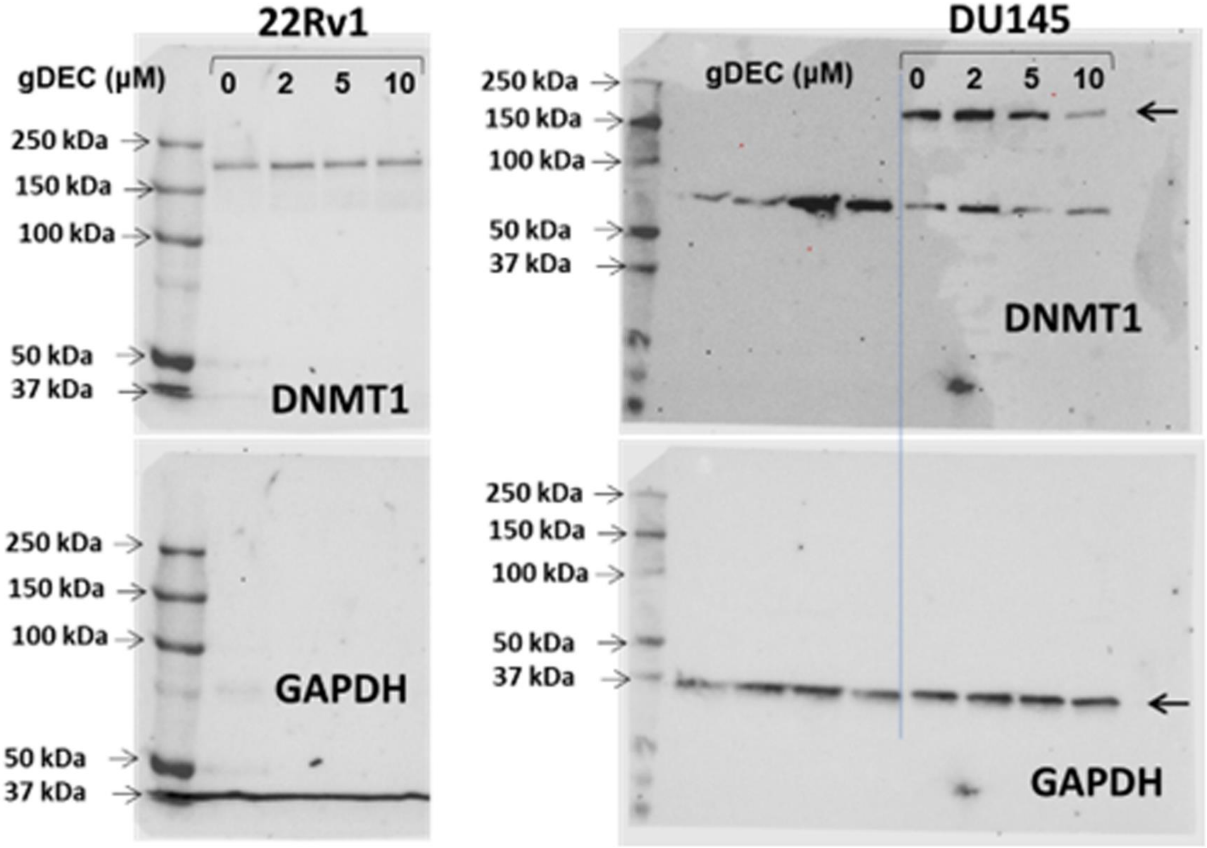

Representative blots: SF 2A

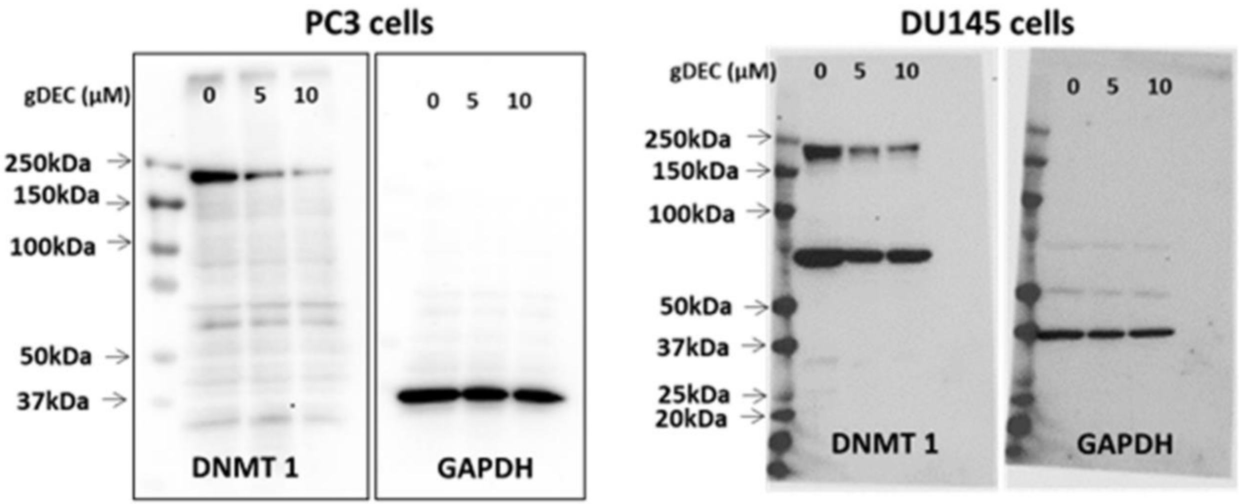

Representative blots of SF 3

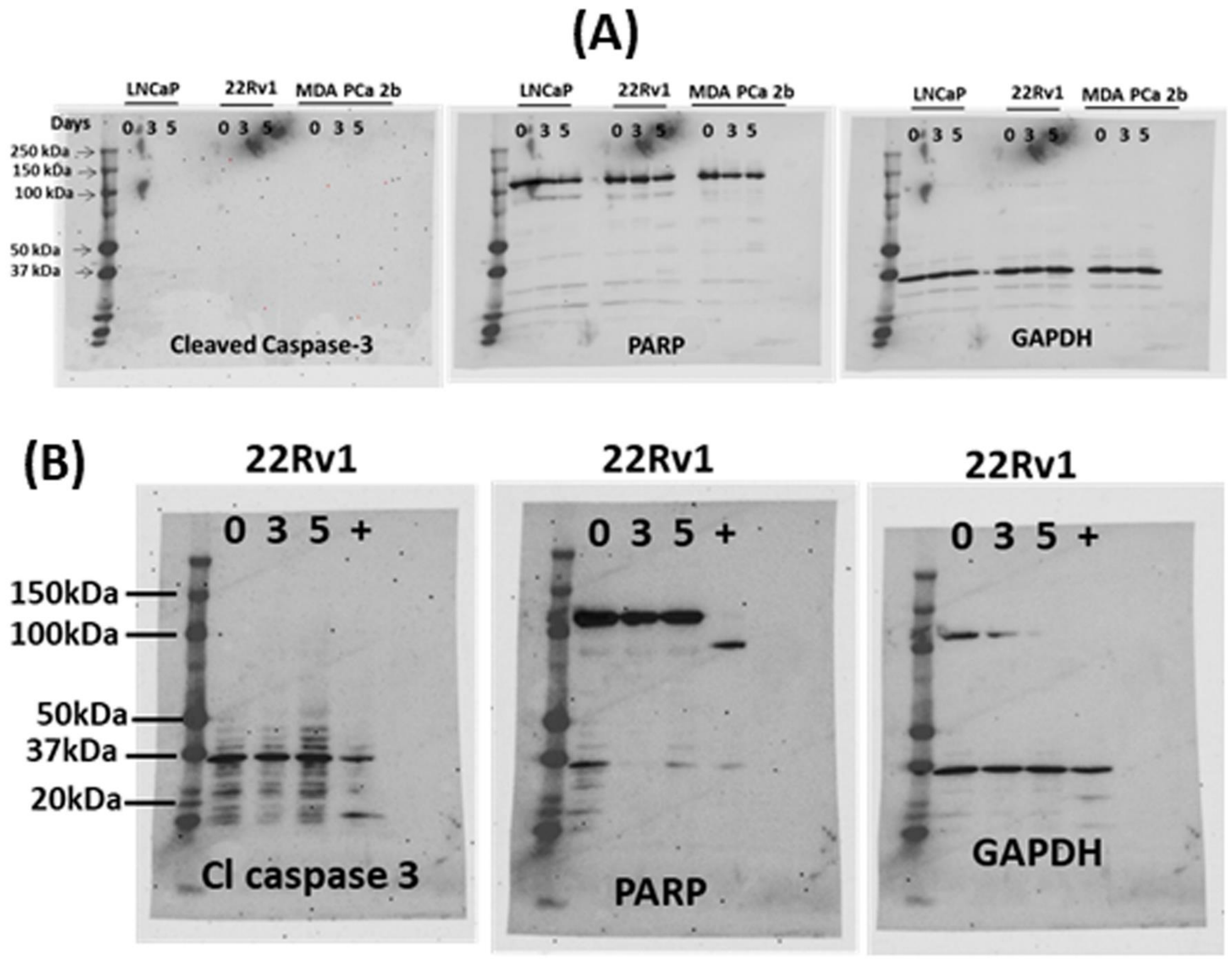

Representative blots: SF5

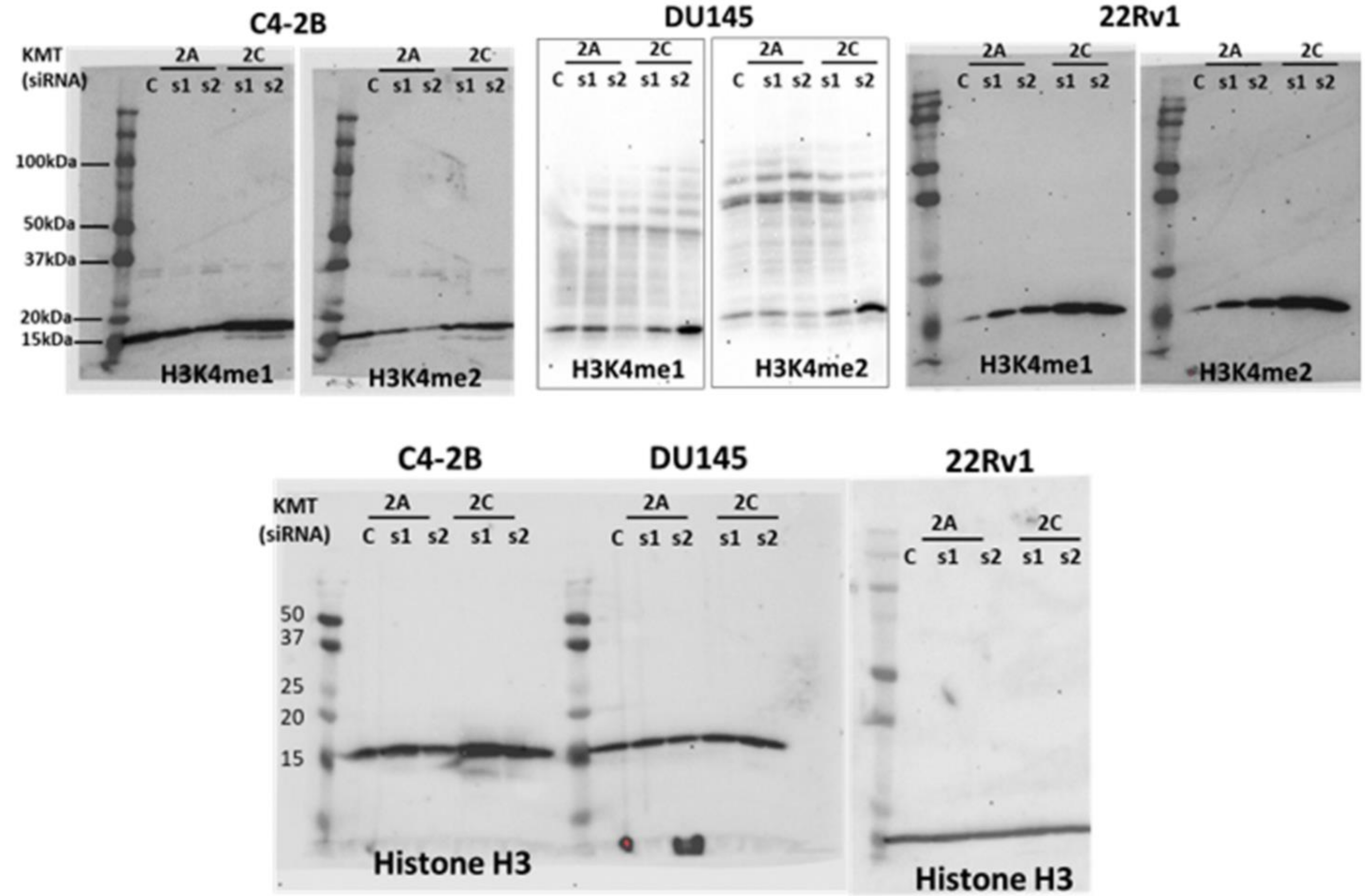

Supplement: Supplementary file 1 [file cancers-15-02763-s001.zip › cancers-2306322-File S1.pdf]
