# Supplementary material for: DNA Methyltransferase 1 Targeting Using Guadecitabine Inhibits Prostate Cancer Growth by an Apoptosis-Independent Pathway
Source: Cancers (Basel). 2023 May 15;15(10):2763. doi: 10.3390/cancers15102763 (PMC10216613; doi:10.3390/cancers15102763)
Supplement: Supplementary file 1 [file cancers-15-02763-s001.zip › cancers-2306322-supplementary.pdf]

Supplementary Figures Legend

Supplementary Figure S1. (**A**) MTT-based **c**ell viability analysis in 22Rv1 prostate cancer cells treated with 0.625 to 25 μM of gDEC, (**B**) Dose titration analysis of gDEC (2, 5, and 10 μM) following 3 day treatment of 22Rv1 and DU145 cells inhibiting DNMT1. Original western blots are presented in File S1.

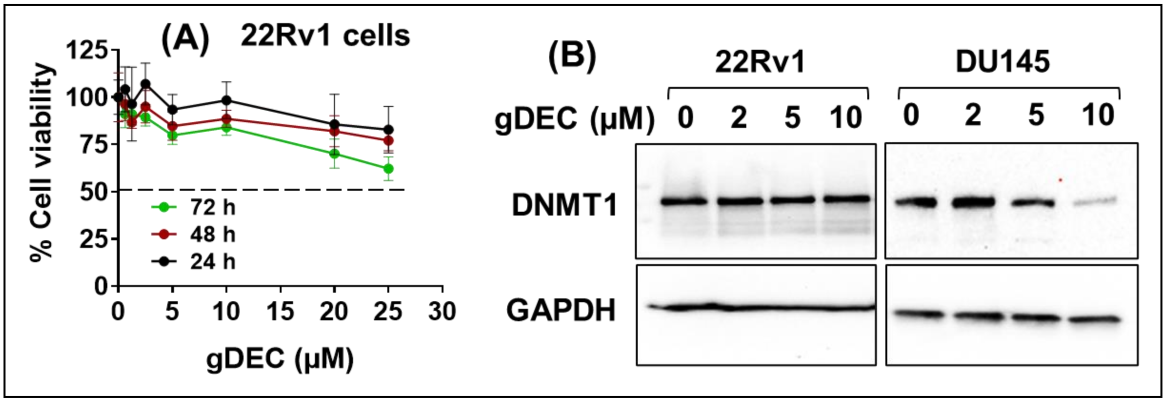

Supplementary Figure S2. Effect of gDEC on PC-3 and DU145 prostate cancer cell lines. (**A**) DNMT1 inhibition, (**B**) Anti cell proliferation, and (**C**) Inhibition of colony formation. Experimental details are the same as detailed in Figures 1 and 2. Significant levels are ***P < 0.001, ****P < 0.0001. Original western blots are presented in File S1.

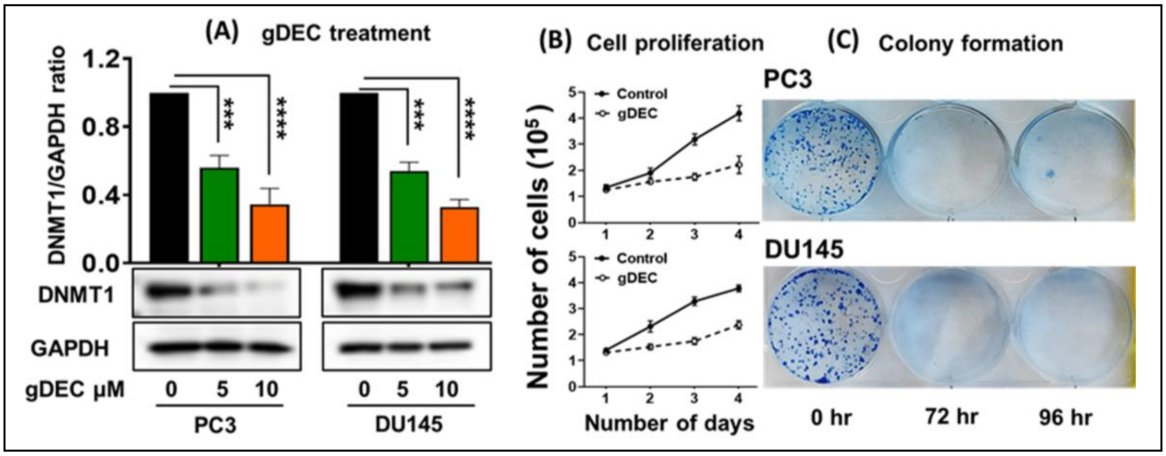

Supplementary Figure S3. Effect of gDEC on apoptosis markers in prostate cancer cells. (**A**) LNCaP, 22Rv1, MDA PCa 2b, and PC-3 cells were treated with 10 µM gDEC for 3- and 5-days, and the protein samples were analyzed for cleaved PARP and caspase-3 expression with GAPDH as a loading control. (**B**) Re-examination of 22Rv1 cells for PARP and caspase-3 cleavage with positive controls validating the effect of gDEC. Original western blots are presented in File S1.

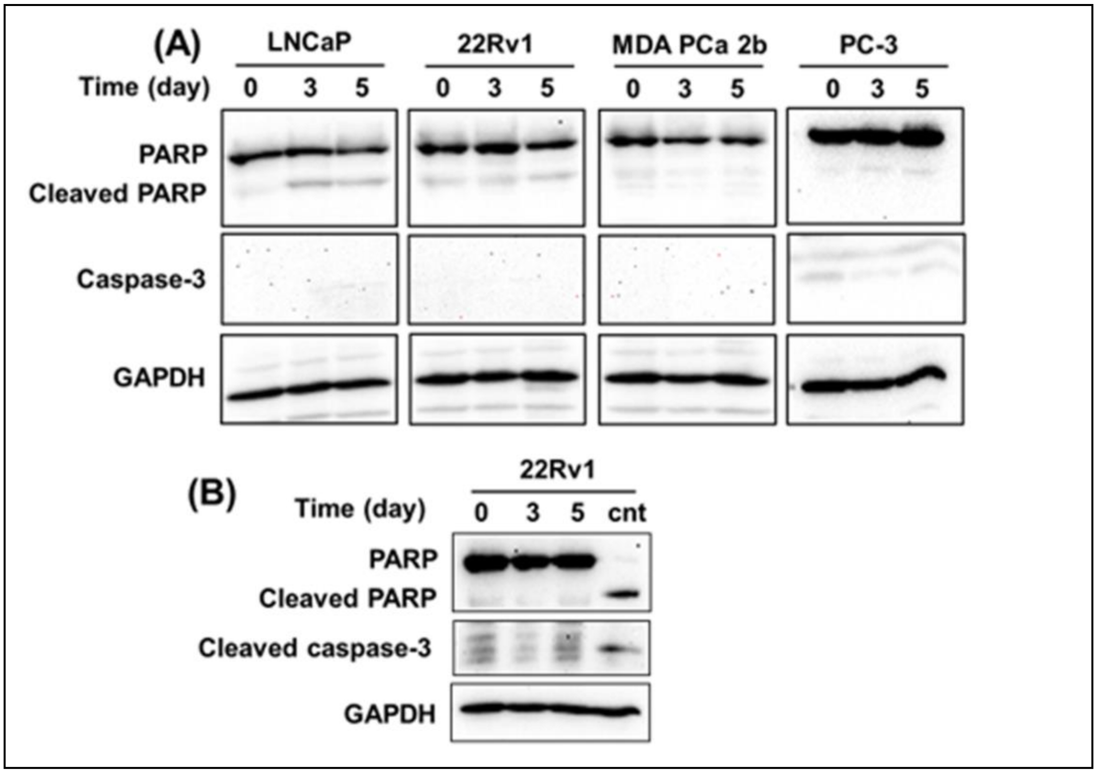

Supplementary Figure S4. Effect of gDEC on **(A)** Tumor weight in 22Rv1 and MDA PCa 2b xenografts. On day 23, the tumor was excised and weighed. **(B)** Body weight in mice challenged with 22Rv1 and PC-3 cells compared to vehicle controls. There was no significant change (22Rv1: $p = 0.763$ and PC-3: $p = 0.242$) in the body weight between control and gDEC-treated animals. Error bar represents mean ± SEM. Significant level is **P < 0.01 for tumor weight.

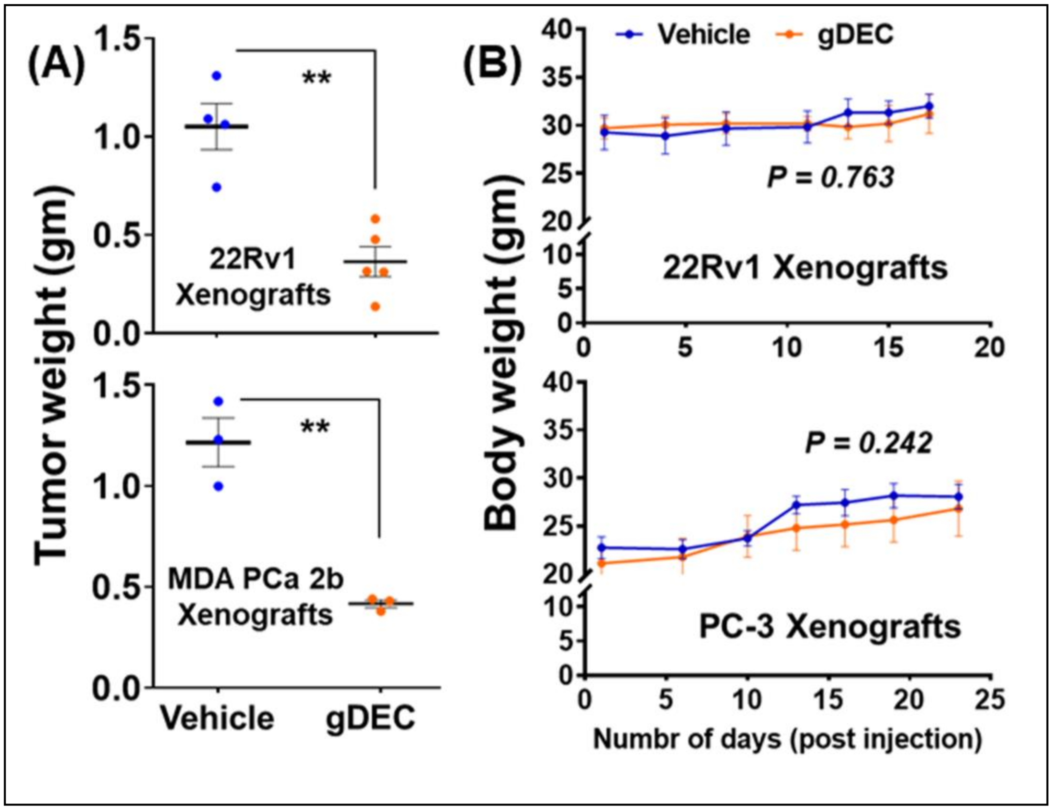

Supplementary Figure S5. (**A**) Analysis of siRNA-directed KMT2A and KMT2C knockdown, and (**B**) effect of KMT2A and KMT2C knockdown on H3K4 methylation. C represents a siRNA scramble control, while s1 and s2 are two different siRNAs for KMT2A and KMT2C. This experiment was repeated 2-3 times from independent cell preparations with similar results. Original western blots are presented in File S1.

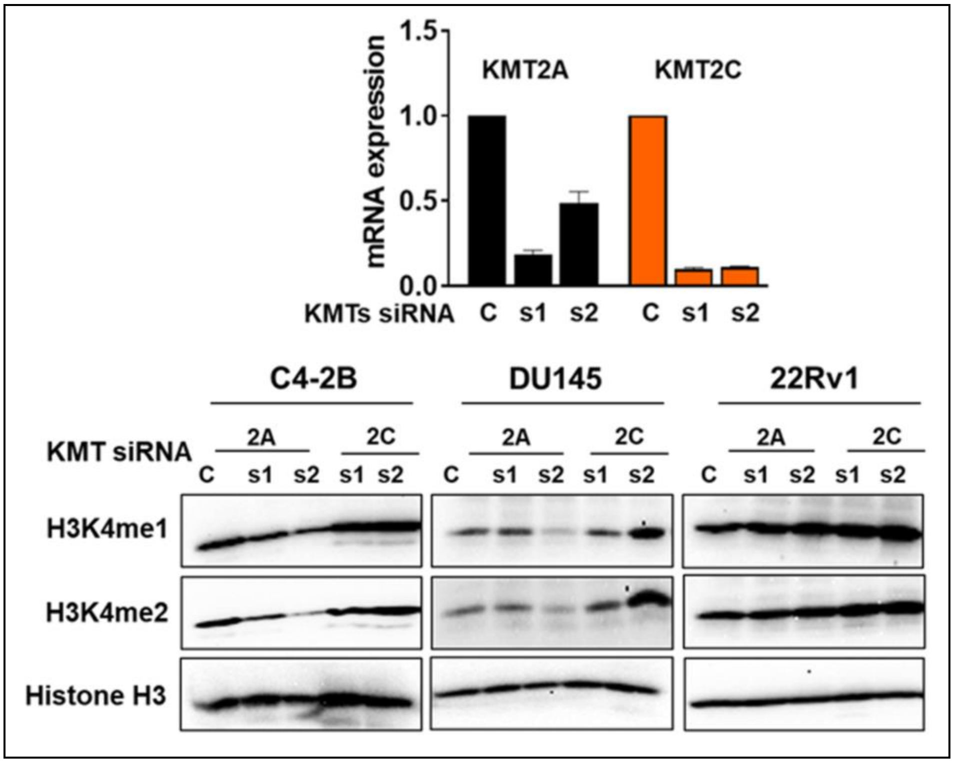

Table S1. List of pre-designed SYBR GREEN primers sequence from Sigma used for RT-qPCR.

Gene name	Forward primer (5' to 3')	Reverse primer (5' to 3')
**KMT2A**	GAGGACCCCGGATTAAACAT	GGAGCAAGAGGTTCAGCATC
**KMT2C**	AAGCAAACGGACTCAGAGGA	ACAAGCCATAGGAGGTGGTG
**KMT2E**	ATCAGGCGGTCTTGTACACC	TCTTTGAGGCATGCACAGTC
**KMT2H**	AAGCTTTAATGAAGCACCAG	TCTGATACTTCTTCTTTGGGG
**KMT5A**	GGAAGGAAGAAGGAATGAAG	GAAAATAGTACATGTAGCAGCC
**E-cadherin**	CCGAGAGCTACACGTTC	TCTTCAAAATTCACTCTGCC
**N-cadherin**	ACATATGTGATGACCGTAAC	TTTTTCTCGATCAAGTCCAG
**GAPDH**	ACAGTTGCCATGTAGACC	TTGAGCACAGGGTACTTTA
